# Supplementary material for: SMAD3 Hypomethylation as a Biomarker for Early Prediction of Colorectal Cancer
Source: Int J Mol Sci. 2020 Oct 7;21(19):7395. doi: 10.3390/ijms21197395 (PMC7582763; doi:10.3390/ijms21197395)
Supplement: Supplementary file 1 [file ijms-21-07395-s001.zip › SMAD3 Hypomethylation As a Biomarker for Early Prediction of Colorectal Cancer (Supplementary Materials - Table ).docx]

**Table S1.** Alterations of *SMAD3* in relation to the clinical parameters of the TCGA CRC dataset

| **Characteristics** | | **Total *n*** | ***SMAD3* methylation**  **Low *n* (%) High *n* (%)** | | | | | **Total *n*** | ***SMAD3* mRNA**  **Low *n* (%) High *n* (%)** | | | | | |  |
| --- | --- | --- | --- | --- | --- | --- | --- | --- | --- | --- | --- | --- | --- | --- | --- |
|  |  |  |  |  |  | | |  |  | |  | | | |  |
| **Overall** | | 279 | 219 | (78.5) | | 60 | (21.5) | 435 | 363 | (83.4) | 72 | (16.6) | |  |  |
| **Age** | |  |  |  |  | |  |  |  |  |  | |  | |  |
|  | < 65 | 124 | 96 | (77.4) | 28 | | (22.6) | 163 | 135 | (82.8) | 28 | | (17.2) | |  |
|  | > 65 | 155 | 123 | (79.4) | 32 | | (20.6) | 272 | 228 | (83.8) | 44 | | (16.2) | |  |
| **Race** | |  |  |  |  | |  |  |  |  |  | |  | |  |
|  | Asian | 10 | 8 | (80.0) | 2 | | (20.0) | 10 | 8 | (80.0) | 2 | | (20.0) | |  |
|  | Black or African American | 52 | 39 | (75.0) | 13 | | (25.0) | 53 | 40 | (75.5) | 13 | | (24.5) | |  |
|  | White | 199 | 160 | (80.4) | 39 | | (19.6) | 209 | 169 | (80.9) | 40 | | (19.1) | |  |
| **Sex** | |  |  |  |  | |  |  |  |  |  | |  | |  |
|  | Male | 150 | 119 | (79.3) | 31 | | (20.7) | 229 | 194 | (84.7) | 35 | | (15.3) | |  |
|  | Female | 129 | 100 | (77.5) | 29 | | (22.5) | 206 | 169 | (82.0) | 37 | | (18.0) | |  |
| **Tumor Type** | |  |  |  |  | |  |  |  |  |  | |  | |  |
|  | Adenocarcinoma | 238 | 187 | (78.6) | 51 | | (21.4) | 373 | 309 | (82.8) | 64 | | (17.2) | |  |
|  | Mucinous | 38 | 29 | (76.3) | 9 | | (23.7) | 57 | 49 | (86.0) | 8 | | (14.0) | |  |
| **Tumor Stage** | |  |  |  |  | |  |  |  |  |  | |  | |  |
|  | I | 42 | 36 | (85.7) | 6 | | (14.3) | 72 | 59 | (81.9) | 13 | | (18.1) | |  |
|  | II | 112 | 90 | (80.4) | 22 | | (19.6) | 175 | 151 | (86.3) | 24 | | (13.7) | |  |
|  | III | 78 | 59 | (75.6) | 19 | | (24.4) | 117 | 94 | (80.3) | 23 | | (19.7) | |  |
|  | IV | 38 | 27 | (71.1) | 11 | | (29.9) | 61 | 51 | (83.6) | 10 | | (16.4) | |  |
| **Tumor Size** | |  |  |  |  | |  |  |  |  |  | |  | |  |
|  | T0-T1 | 7 | 7 | (100) | 0 | | (0) | 11 | 11 | (100) | 0 | | (0) | |  |
|  | T2-T4 | 271 | 211 | (77.9) | 60 | | (22.1) | 423 | 352 | (83.2) | 71 | | (16.8) | |  |
| **Regional lymph nodes metastasis** | | | |  |  | |  |  |  |  |  | |  | |  |
|  | N=0 | 164 | 131 | (79.9) | 33 | | (20.1) | 260 | 220 | (84.6) | 40 | | (15.4) | |  |
|  | N>1 | 115 | 88 | (76.5) | 27 | | (23.5) | 175 | 143 | (81.7) | 32 | | (18.3) | |  |
| **Distant metastasis** | | | |  |  | |  |  |  |  |  | |  | |  |
|  | M=0 | 188 | 153 | (81.4) | 35 | | (18.6) | 319 | 266 | (83.4) | 53 | | (16.6) | |  |
|  | M>1 | 38 | 27 | (71.1) | 11 | | (28.9) | 61 | 51 | (83.6) | 10 | | (16.4) | |  |
| **MSI** | |  |  |  |  | |  |  |  |  |  | |  | |  |
|  | No | 72 | 63 | (87.5) | 9 | | (12.5) | 76 | 66 | (86.8) | 10 | | (13.2)^0.020^ | |  |
|  | Yes | 11 | 11 | (100) | 0 | | (0) | 11 | 6 | (54.5) | 5 | | (45.5) | |  |
| **Kras mutation** | |  |  |  |  | |  |  |  |  |  | |  | |  |
|  | No | 258 | 199 | (77.1) | 59 | | (22.9) | 413 | 342 | (82.8) | 71 | | (17.2) | |  |
|  | Yes | 21 | 20 | (95.2) | 1 | | (4.8) | 22 | 21 | (95.5) | 1 | | (4.5) | |  |
|  | |  |  |  |  | |  |  |  |  |  | |  | |  |
|  |  |  |  |  |  | |  |  |  |  |  | |  | |  |
|  | 1. These results were analyzed based on the Pearson's *X^2^* test. Significant *p* values are indicated by superscripts. For some categories, the number of samples (*n*) was lower than the overall number analyzed because clinical data were unavailable for these samples. 2. When the *SMAD3* methylation level (ΔAvg_β), as determined with the Illumina Infinium HumanMethylation450 BeadChip array, in CRC tumors from the TCGA dataset was lower than 0.1, a sample was considered to have hypomethylation. 3. When the *SMAD3* expression level determined by RNA sequencing analysis of CRC tumors from the TCGA dataset was less than the median *SMAD3* expression level in adjacent normal colon tissues, a sample was considered to have low expression. | | | | | | | | | | | | | | |
