# Supplementary material for: SMAD3 Hypomethylation as a Biomarker for Early Prediction of Colorectal Cancer
Source: Int J Mol Sci. 2020 Oct 7;21(19):7395. doi: 10.3390/ijms21197395 (PMC7582763; doi:10.3390/ijms21197395)
Supplement: Supplementary file 1 [file ijms-21-07395-s001.zip › SMAD3 Hypomethylation As a Biomarker for Early Prediction of Colorectal Cancer (Supplementary Materials - Figures).docx]

**Supplementary Materials**

**
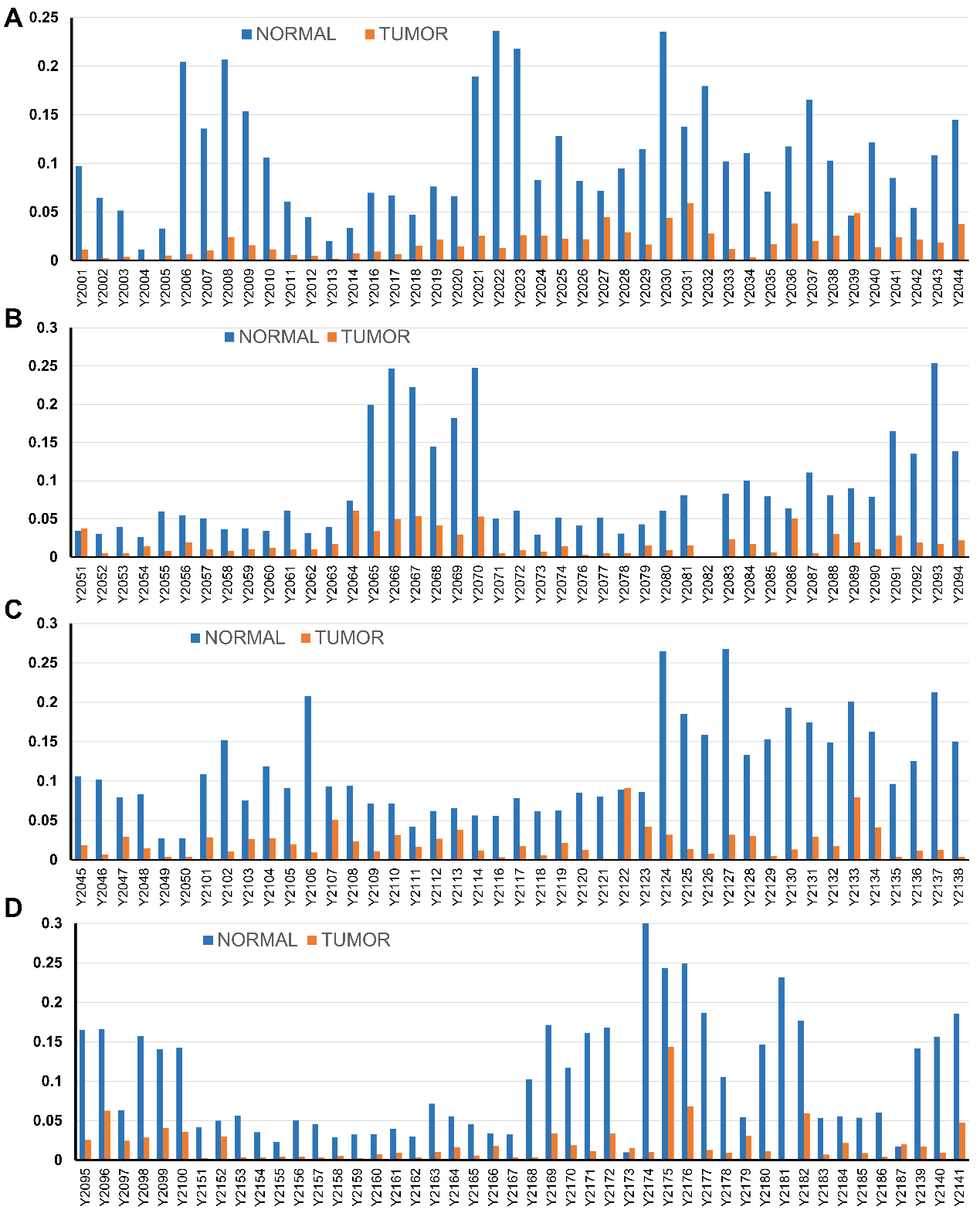
**

**
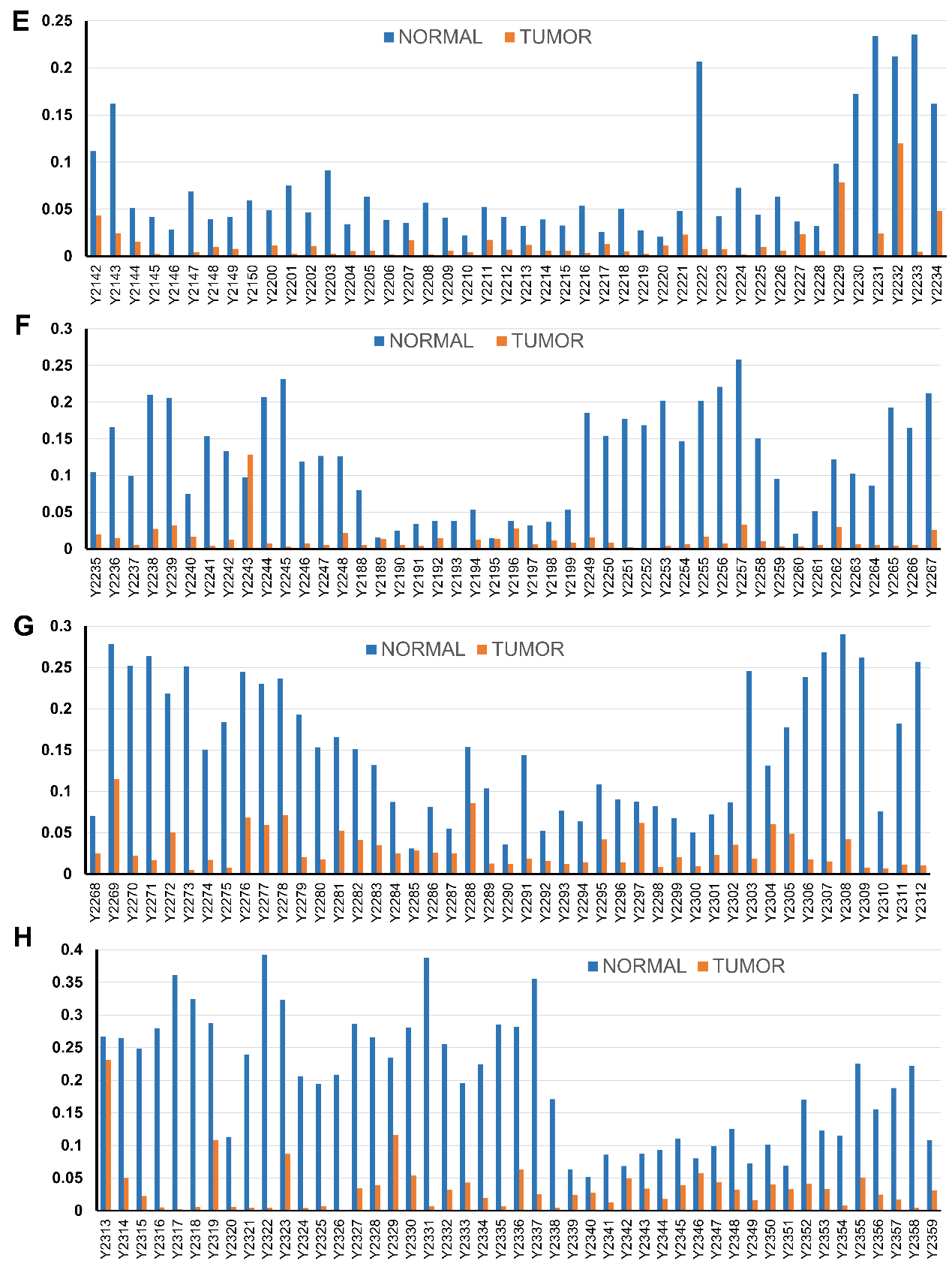
**

**
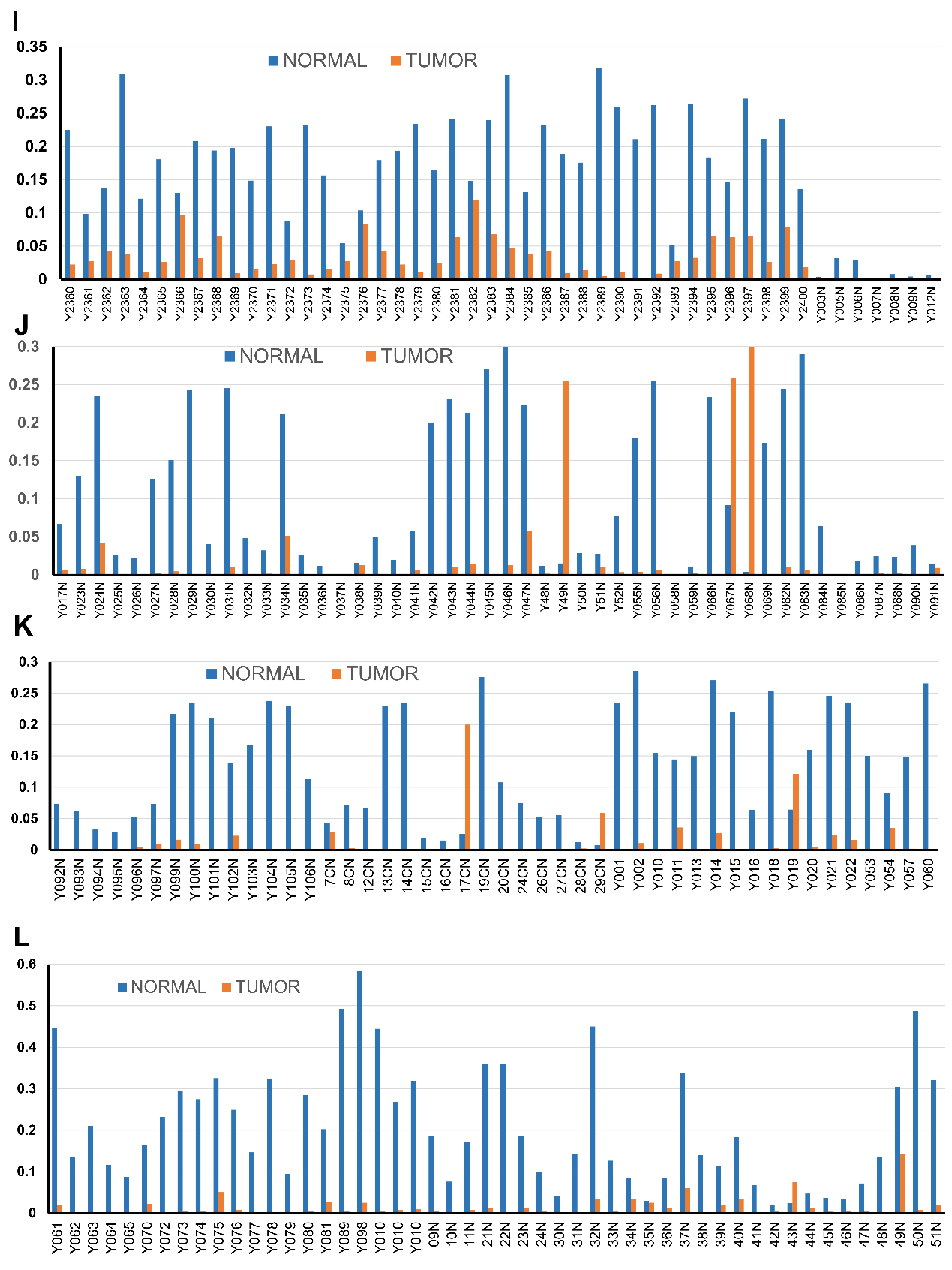
**

**Figure S1. *SMAD3* methylation level in Taiwanese colorectal cancer patients:** (A)-(L) Levels of methylated *SMAD3* were determined using quantitative methylation-specific polymerase chain reaction (QMSP) in tumor tissue and adjacent normal tissue samples from 548 Taiwanese patients with CRC. ACTB was used as the internal control


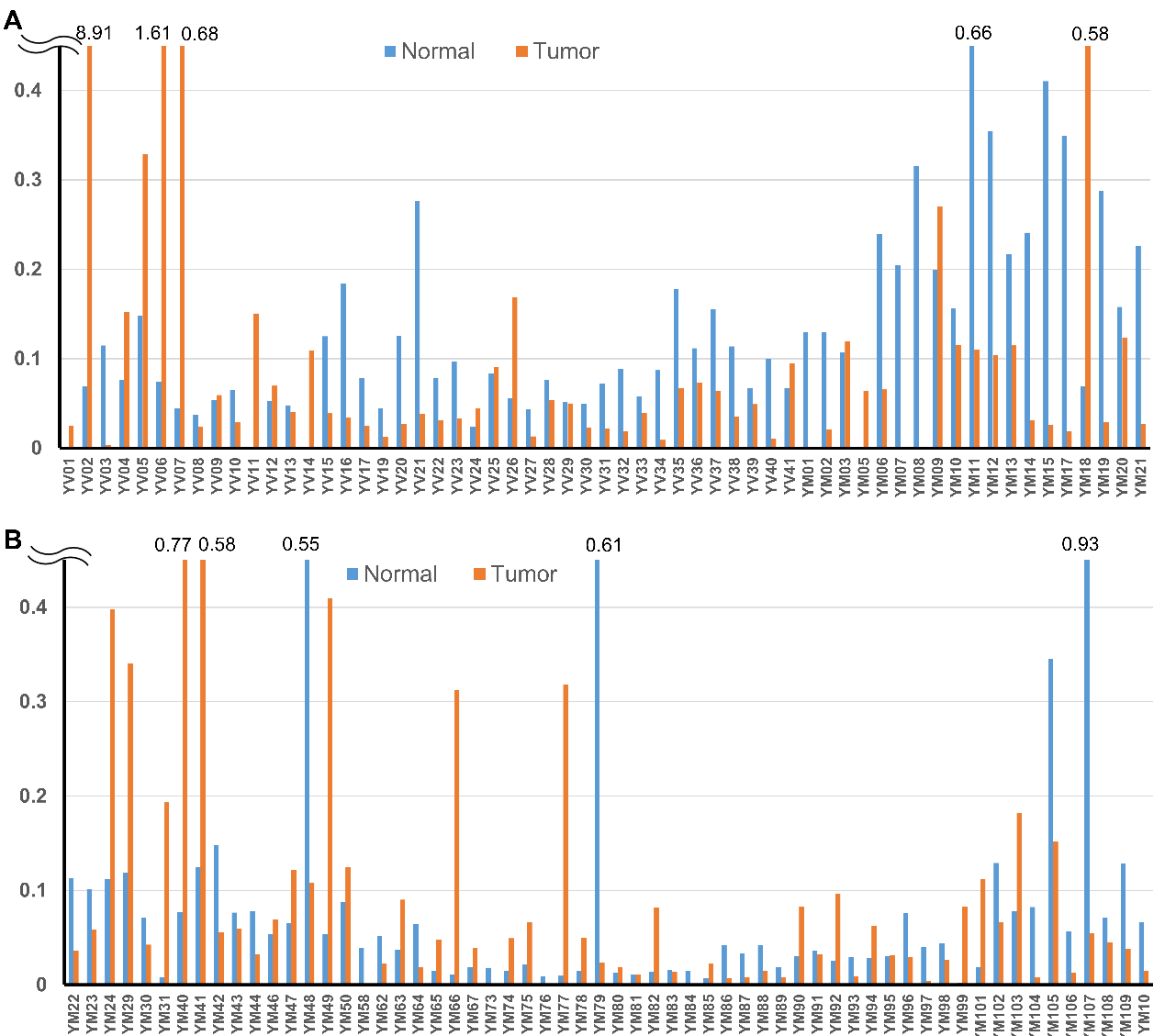


**Figure S2. The mRNA expression level of *SMAD3* in Taiwanese patients with CRC:** (A)–(B) The *SMAD3* mRNA expression level was determined using quantitative reverse transcription–polymerase chain reaction (RT–PCR). Tumor and adjacent normal tissue samples were obtained from 119 patients. GAPDH was used as the internal control.


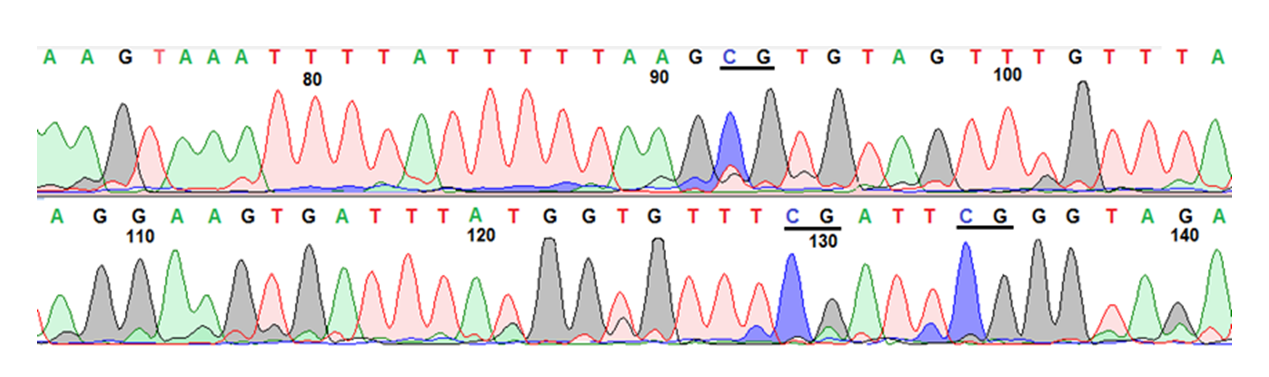


**Figure S3. Hypomethylation of the *SMAD3* was analyzed in CRC patients:** The specificity of *SMAD3* methylation end products was confirmed by bisulfite sequencing.
